# Effectiveness of novel facial stretching with structured exercise versus conventional exercise for Bell’s palsy: a single-blinded randomized clinical trial

**DOI:** 10.1038/s41598-024-64046-z

**Published:** 2024-06-10

**Authors:** Stephanie Santiago, Abraham M. Joshua, Akshatha Nayak, Zulkifli Misri, Shivananda Pai, Rohit Pai, Prasanna Mithra, Ashish John Prabhakar, Vijayakumar Palaniswamy

**Affiliations:** 1 Department of Physiotherapy, Kasturba Medical College, Mangalore, Manipal Academy of Higher Education, Manipal, India; 2 Department of Neurology, Kasturba Medical College, Mangalore, Manipal Academy of Higher Education, Manipal, India; 3 Department of Community Medicine, Kasturba Medical College, Mangalore, Manipal Academy of Higher Education, Manipal, India

**Keywords:** Bell’s palsy, Facial exercises, Therapy, Rehabilitation, Facial symmetry, Disability, Diseases of the nervous system, Motor control, Neuroscience, Neurology, Clinical trial design

## Abstract

The purpose of the study was to assess the effects of a novel technique involving facial stretching of the unaffected side along with a structured exercise for the affected side on facial symmetry and facial functions as compared to conventional exercise. A hospital-based parallel-group randomized trial was completed among patients with acute Bell’s palsy in Mangalore, India. Participants were randomized to receive facial stretching and a structured exercise program (experimental group) or the conventional exercise regimen (conventional group). Primary outcomes were facial symmetry and voluntary movement; assessed by the Sunnybrook Facial Grading System (SFGS). Both regimens were given for 3 weeks, with baseline, 10th day, and 20th day assessments. Out of 31 participants screened, 24 were eligible and 12 participants each were assigned to experimental and conventional groups. Change scores revealed greater improvement in the SFGS score (*p* = 0.002) for the experimental group participants. Facial stretching and structured exercise program exhibited promising results in enhancing facial symmetry and function in acute Bell’s palsy when compared to conventional exercise regimen.

## Introduction

Bell’s palsy (BP) is an acute primary idiopathic lower motor neuron disorder characterized by sudden unilateral facial weakness^1^, with an annual incidence ranging from 11.5 to 53.5 per 100,000 persons^2,3^. The symptoms of BP can range from mild weakness to severe paralysis and include, absence of forehead wrinkling, drooping of the mouth corner, eye dryness or excessive tearing, ear pain, difficulty eating, and impaired taste sensation^4^. While about two-thirds of patients recover fully within three months, a significant proportion continue to experience persistent symptoms, including facial asymmetry and involuntary movements (synkinesis) which impact their psychological and social quality of life^4–6^.

Current medical management of BP primarily involves antivirals and corticosteroids. A 2019 Cochrane review suggested limited effectiveness of these interventions in resolving BP symptoms^7^. However, a 2020 systematic review indicated potential benefits of this combination in reducing long-term sequelae^8^.

Physical therapy interventions for BP include facial muscle expression exercises, facial massage, electrotherapy, diathermy, kinesiotaping, and neuromuscular reeducation^4–6,9,10^. A recent systematic review demonstrated the clinical effectiveness of facial muscle exercises in enhancing early recovery of facial muscle strength, symmetry, and movement, as indicated by Sunnybrook Facial Grading System (SFGS) scores, and overall facial function, reflected in Facial Disability Index (FDI) scores. This study compared facial muscle exercises with various other interventions, including electrical stimulation, mirror biofeedback, acupuncture, mime therapy, Proprioceptive Neuromuscular Facilitation (Kabat), antiviral and corticosteroid drugs, hyperbaric oxygen therapy, facial re-training, surgical interventions, laser therapy, taping techniques, feedback training, and combination therapies^11^. Conversely, a prior systematic review examining diverse physical therapy interventions for BP suggested that while facial exercises may alleviate symptoms, particularly in acute cases, their efficacy for idiopathic facial paralysis lacks robust evidence and necessitates further high-quality research^12^.

Current physiotherapy interventions primarily target the primary impairment on the paretic side^13,14^. However, the muscle imbalance resulting from facial palsy, which places the non-paretic side muscles in a perpetually shortened state, is never addressed in previous studies. This shortened state can cause architectural changes at the functional unit and connective tissue levels including a reduction of sarcomeres in series, and passive stiffness and/or contracture development in intramuscular connective tissues^15,16^. Additionally, overactivity of the non-paretic side muscles perpetually elongates/stretches the paretic muscles beyond their optimal length, limiting their force generation capacity^17^. However, these changes can be prevented by passive stretching or active stimulation^18^. In particular, short, daily periods of stretch can prevent the loss of sarcomeres and muscle atrophy^19^.

Given the conflicting evidence and limitations of current physical therapy approaches, this study introduces a novel facial stretching technique combined with a structured exercise program. This combined stretching and strengthening exercise program, grounded in systematic review findings and previous studies^12,20,21^, highlights the importance of addressing facial muscle imbalances. Such an approach promotes facial symmetry by reducing overactive unaffected side muscles and strengthening the weaker side with structured exercises^22^. Muscle balance is essential for harmonious facial movements^23^ and the current study attempts to assess the efficacy of a novel stretching technique combined with a structured exercise program against conventional therapy for improving facial asymmetry, synkinesis, and facial neuromuscular function among acute BP patients.

## Methods

### Study design

The parallel-group facility-based randomized clinical trial was conducted in the hospitals attached to Kasturba Medical College, Mangalore. Approval was obtained from the Institutional Ethics Committee (IEC KMC MLR-11-19/575). The trial was registered in the Clinical Trials Registry India (CTRI/2020/02/023,240) on 11/02/2020. The study adhered to the ethical principles of the Declaration of Helsinki for research involving human participants.

### Participants

The study included participants with acute, primary unilateral idiopathic lower motor neuron facial palsy (Bell’s Palsy). The exclusion criteria comprised of secondary lower motor neuron facial palsy, temporomandibular joint dysfunction, prosthetic dentures or braces, facial dysmorphism, and severe ear pain potentially interfering with assessment or treatment.

The sample size was calculated using the below-mentioned formula with an assumption of a difference in the change score of 20 for facial symmetry and function across the two intervention arms, 90% power, a clinically accepted difference of 15, and a 10% non-response rate, resulting in 12 participants in each study arm.

$$N = { 2 } \times \frac{{(Z{1} - \alpha + Z{1} - \beta )^{{2}} }}{\partial - \partial 0} \times s{2}$$

where *Z*1 − α = 1.96, *Z*1 − β = 1.281.

Participants were enrolled from February 2020 to March 2021, following referrals from neurologists or physicians. Prior to enrollment, informed consent was obtained from each interested participant who met the eligibility criteria.

### Medical management

All participants were treated according to the National Institutes of Health (NIH) guidelines for acute BP, as recommended in previous studies^10,24^. The medical regimen consisted of Prednisone at a dose of 60 mg daily for the initial 5 days, followed by a tapering dose of 10 mg per day over the subsequent 5 days. Additionally, participants received oral Acyclovir or Valacyclovir for the first 5 days, aligning with established protocols^4^.

### Randomization and masking

Participants were randomly assigned to experimental group (facial stretching and structured exercise regimen) or the conventional group (conventional exercise regimen) using a permuted block randomization technique with varying block (4 and 6). Allocation concealment was maintained using the opaque envelop method with the sequence secured by one investigator (PM). Another investigator (AN) assigned participants to the study groups.

### Interventions

Both groups received therapy 6 days a week for 3 weeks. Each session began with a faradic galvanic test to gauge facial muscle response irrespective of the intervention arm. Responsive participants received faradic current targeting facial muscles in two sets of 30 contractions while non-responsive participants received galvanic stimulation over the facial nerve trunk and motor branches. Both groups received only one session of faradic/galvanic stimulation per day.

The experimental group received four stretching techniques: three on the unaffected side and one on the affected side of face. During initial sessions, bystanders or family members were trained in three techniques through hands-on guidance from the therapist and were given printed instructional handouts. All four stretching techniques were repeated 10 times with a stretch hold of 10–15 s. The patient was directly taught the fourth technique for the affected side. They were instructed to perform the stretching technique four sessions daily at home. Follow-up assessments ensured that the correct technique was executed. Following each session’s stretching exercise, participants in the experimental group performed a specific set of exercises that focused on minimizing or avoiding overactivity of the facial muscles on the unaffected side.

Participants in the conventional group received exercise based on the Northeast London Foundation Trust (NELFT)-National Health Service (NHS) guidelines. Conventional group participants were instructed to perform these exercises four times daily at home, with follow-up assessments to ensure correct execution. Both groups were instructed to perform facial exercises in front of the mirror. Details of both experimental and conventional exercise protocols are appended and accessible at 10.17605/OSF.IO/UVW89. Models have provided informed consent for the publication of animated images included in the online open access appendix.

### Outcome measures

The primary outcome measure was the Sunnybrook Facial Grading System (SFGS), a regional weighted system assessing resting symmetry, voluntary movement, and synkinesis. The total SFGS composite score ranges between 0 and 100, where 0 stands for total paralysis and 100 for normal facial function. The House Brackmann Scale (HBS), a widely accepted facial grading system, was included as a secondary measure to evaluate the severity of nerve damage in facial nerve palsy. Additionally, the Facial Disability Index (FDI), consisting of physical and social subscales, was included to assess the disability related to facial neuromuscular dysfunction. Using standard procedures, blinded independent assessors performed assessments at baseline, on the 10th day (mid-intervention), and on the 20th day (post-intervention).

### Statistical analysis

Data analysis was performed using IBM SPSS Statistics Version 25.0. Demographic and clinical characteristics at baseline were compared between treatment groups to assess the effectiveness of the randomization procedure. Results were presented as proportions and summary measures. Group comparisons utilized the Kruskal–Wallis, Wilcoxon’s signed-rank, and Mann–Whitney U tests. Analyses of outcome data were performed by an experienced statistician blinded to group allocation. A *p*-value of < 0.05 was considered statistically significant.

## Results

**Figure 1:**
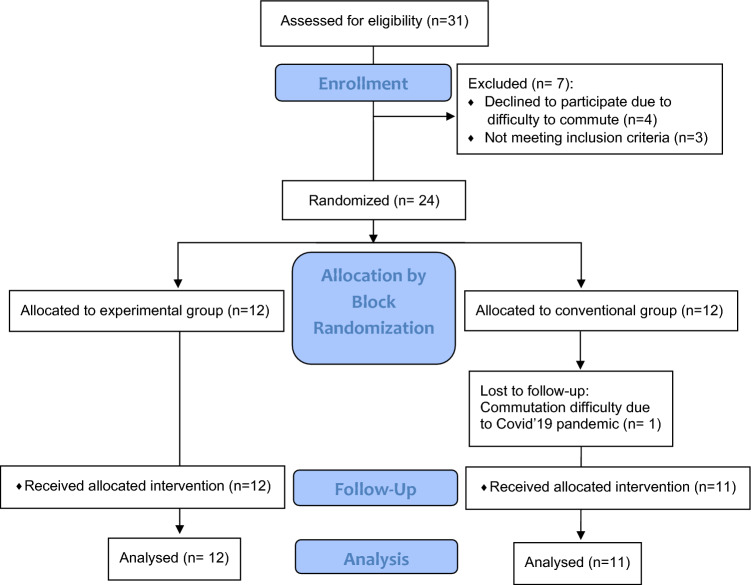
Consort flow chart.

Thirty-one study participants underwent eligibility assessment, of which three were excluded based on inclusion and exclusion criteria and four due to difficulty commuting. The CONSORT flow chart is shown in Fig. 1. Of the 24 eligible patients, 12 were randomized to each group: facial stretching and exercise or conventional exercise. Following allocation to the conventional group, due to travel ban imposed during the COVID19 pandemic, data collection for one patient was incomplete and lost to follow-up. Demographic details and baseline characteristics for the remaining 23 participants are provided in Table 1. Treatment compliance rates were 100% for the experimental group and 91.6% for the conventional group.

**Table 1:** Baseline characteristics of the study groups.

Variables	Experimental group (n = 12) median (IQR)	Conventional group (n = 11) median (IQR)	*p-*value
Age [Years]	39.5 (33.5–50.8)	45.0 (35.0–50.0)	0.74
Gender			
Male (%)	58.3	90.9	0.16
Female (%)	41.7	09.1	
SFGS-Resting asymmetry	15.0 (11.3–15.0)	15.0 (05.0–15.0)	0.57
SFGS-Voluntary movement	46.0 (37.0–69.0)	44.0 (36.0–56.0)	0.69
SFGS-Total composite score	31.5 (22.0–57.0)	31.0 (25.0–41.0)	0.97
FDI-physical function	63.25 (55.0–81.1)	71.5 (49.5–93.5)	0.49
FDI- social function	102.0 (76.0–115.0)	80.0 (72.0–112.0)	0.41

IQR, Inter Quartile Range; n, Number; SFGS, Sunnybrook Facial Grading System; FDI, Facial Disability Index.

Overall, there were no significant differences in resting asymmetry, voluntary movement, and total composite score on the SFGS at baseline between the groups. Baseline, mid, and post-intervention values of SFGS and FDI are presented in Table 2.

**Table 2:** Baseline, mid and post intervention values of SFGS and FDI.

Outcome measures	Time period	Experimental group (n = 12) [Median (IQR)]	Conventional group (n = 11) [Median (IQR)]	*p*-value
SFGS-resting asymmetry	Baseline	15.0 (11.3–15.0)	15.0 (05.0–15.0)	0.566
	10th day	05.0 (00.0–10.0)	15.0 (05.0–15.0)	0.134
	20th day	00.0 (00.0–00.0)	10.0 (00.0–15.0)	0.013*
	*p*-value	< 0.0001*	0.015*	
SFGS-voluntary movement	Baseline	46.0 (37.0–69.0)	44.0 (36.0–56.0)	0.695
	10th day	70.0 (59.0–89.0)	48.0 (40.0–76.0)	0.079
	20th day	90.0 (82.0–100.0)	60.0 (52.0–88.0)	0.016*
	*p*-value	< 0.0001*	< 0.0001*	
SFGS-total composite score	Baseline	31.5 (22.0–57.0)	31.0 (25.0–41.0)	0.976
	10th day	62.0 (56.5–89.0)	39.0 (33.0–68.0)	0.079
	20th day	90.0 (80.7–100.0)	50.0 (39.0–84.0)	0.013*
	*p*-value	< 0.0001*	< 0.0001*	
FDI physical function	Baseline	63.25 (55.0–81.1)	71.5 (49.5–93.5)	0.487
	10th day	77.0 (67.3–99.0)	82.5 (60.5–99.0)	0.608
	20th day	104.5(94.9–110.0)	99.0 (82.5–110.0)	0.566
	*p*-value	< 0.0001*	< 0.0001*	
FDI social function	Baseline	102.0 (76.0–115.0)	80.0 (72.0–112.0)	0.413
	10th day	114.0 (92.0–116.0)	96.0 (80.0–116.0)	0.347
	20th day	120.0 (113.0–120.0)	108.0 (88.0–116.0)	0.023*
	*p*-value	< 0.0001*	< 0.0001*	

*Statistically significant; n, Number; IQR, Inter Quartile Range; SFGS, Sunnybrook Facial Grading System; FDI, Facial Disability Index.

For resting asymmetry, post hoc analysis showed significant improvement from baseline to the 10th day in the experimental group (*p* = 0.004) compared to the exercise group (*p* = 0.18). Both groups improved significantly between the 10th and 20th days (experimental: *p* = 0.026; conventional: *p* = 0.046). Comparing baseline to 20th day, the experimental group showed higher significance (*p* = 0.001) than the conventional group (*p* = 0.034).

Regarding voluntary movement, the post hoc analysis revealed significant improvement from baseline to 10th day for both groups (experimental: *p* = 0.002; conventional: *p* = 0.007). Improvement continued from 10 to 20th day (experimental: *p* = 0.002; conventional: *p* = 0.005). The experimental group showed higher significance when compared to the exercise group from baseline to 20th day (*p* = 0.002 vs *p* = 0.003). For the total composite score, both groups improved significantly from baseline to the 10th day (experimental: *p* = 0.002; conventional: *p* = 0.005) and the 10th to the 20th day (experimental: *p* = 0.002; conventional: *p* = 0.003). When comparing baseline to the 20th day, the experimental group again showed higher significance (*p* = 0.002 vs *p* = 0.003).

Regarding the physical function of FDI, significant improvement was observed from baseline to 10th day for both groups (experimental: *p* = 0.002; conventional: *p* = 0.011). Improvement continued from the 10th to the 20th day (experimental: *p* = 0.002; conventional: *p* = 0.007). Comparing baseline to the 20th day, both groups showed significance (experimental: p = 0.002; conventional: *p* = 0.005). Post hoc analysis of the FDI’s social function subscale showed significance from baseline to the10th day for both groups (experimental: *p* = 0.012; conventional: *p* = 0.005). From the 10th to the 20th day, only the experimental group revealed significant improvement (*p* = 0.002), while the conventional group did not (*p* = 0.06). When comparing baseline to 20th day, the experimental group had higher significance (*p* = 0.002 vs *p* = 0.005).

Change score comparisons (Table 3) showed significance in resting asymmetry from baseline to mid (*p* = 0.004) and post (*p* = 0.001). Voluntary movement differences were significant from baseline to mid (*p* = 0.023), mid to post (*p* = 0.044), and baseline to post (*p* = 0.013). Total composite score changes were significant from baseline to mid (*p* = 0.003) and post (*p* = 0.002).

**Table 3:** Comparison of change scores across the groups (n = 23).

Outcome measures	Variables	Experimental Group [Median (IQR)]	Conventional Group [Median (IQR)]	*p*-value
SFGS-resting asymmetry	Baseline to 10th day	− 05.0 [(− 13.7)–(− 5.0)]	00.0 (00.0–00.0)	0.004*
	10–20th day	− 02.5 [(− 10.0)–(0.0)]	00.0 [(− 05.0)–(00.0)]	0.316
	Baseline to 20th day	− 15.0 [(− 15.0)–(− 7.5)]	0.0 [(− 05.0) − (0.0)]	0.001*
SFGS-voluntary movement	Baseline to 10th day	24.0 (20.0–28.0)	08.0 (04.0–20.0)	0.023*
	10–20th day	20.0 (08.0–27.0)	12.0 (04.0–16.0)	0.044*
	Baseline to 20th day	48.0 (31.0–48.0)	24.0 (04.0–32.0)	0.013*
SFGS-total composite score	Baseline to 10th day	30.5 (24.25–36.5)	8.0 (4.0–20.0)	0.003*
	10–20th day	26.5 (8.0–34.5)	12.0 (5.0–17.0)	0.104
	Baseline to 20th day	59.0 (42.5–63.0)	24.0 (9.0–32.0)	0.002*
FDI physical function	Baseline to 10th day	11.0 (11.0–16.5)	11.0 (00.0–22.0)	0.379
	10–20th day	22.0 (11.0–31.6)	11.0 (05.5–27.5)	0.051
	Baseline to 20th day	35.7 (27.5–48.1)	16.5 (11.0–38.5)	0.044*
FDI social function	Baseline to 10th day	06.0 (00.0–15.0)	08.0 (04.0–24.0)	0.449
	10–20th day	04.0 (04.0–10.0)	00.0 (00.0–12.0)	0.134
	Baseline to 20th day	10.0 (05.0–20.0)	08.0 (04.0–28.0)	0.880

*Statistically significant; n, Number; IQR, Inter Quartile Range; SFGS, Sunnybrook Facial Grading System; FDI, Facial Disability Index.

For physical function, a significant difference was found from baseline to post (*p* = 0.044) between the groups. No significant change was observed in social function between the groups (*p* > 0.05).

Grading according to the HBS showed 100% improvement in the experimental group and 72.7% in the conventional group by the 20th day.

## Discussion

The current randomized controlled trial aimed to evaluate the effects of a novel treatment technique on facial symmetry and function in participants with acute Bell’s Palsy, as opposed to the conventional exercise regimen. Bell’s Palsy is marked by facial asymmetry due to overactive unaffected muscles and weakened affected muscles from loss of tone, impacting physical, social, and psychological well-being^25–27^. Prior physical therapies often overlooked the muscular imbalance and our unique technique addressed this by stretching overactive muscles and targeting weak muscles with specific strengthening exercises.

Our results exhibited improvements in facial symmetry and function for either group, with the experimental group revealing superior and faster recovery compared to the conventional group. While the conventional group showed minimal improvement, the participants in the combined novel stretching and structured exercises group demonstrated a significant reduction in resting asymmetry from baseline to mid-intervention (experimental: *p* = 0.004; conventional: *p* = 0.18) and further significant improvements to the end of the intervention (experimental: *p* 0.026; conventional: *p* = 0.046). Significant improvements were also observed in voluntary movement and total composite scores in the experimental group during the early stages, with both groups showing improvements over time. In terms of facial functions assessed using the physical subscale of the Facial Disability Index (FDI), the experimental group (*p* = 0.002) demonstrated faster improvement compared to the conventional group (*p* = 0.011). Both groups revealed similar levels of improvement for the social subscale.

The experimental group consistently showed higher significance levels in change scores for resting asymmetry, voluntary movement, and total composite score (*p* < 0.05). This can be attributed to the targeted approach of the novel intervention, which aimed to address both overactive and weakened facial muscles through unique stretching and specific exercise regimen. Evidence from a previous study suggests that targeted exercises can enhance neuromuscular coordination and muscle strength, leading to improved facial symmetry^28^.

Visual inspection of the HBS revealed improvement in all study participants in the experimental group, while some participants in the conventional group showed no improvement. The combined stretching and structured exercise group also had a higher percentage of participants showing improvement compared to the conventional group. Overall, the novel technique showed promising results in enhancing facial symmetry and function in acute BP, potentially preventing synkinesis and improving quality of life. Given the observed patterns of improvement in facial symmetry and function in this study, further research could explore the potential applicability of these findings to a broader population of BP patients with similar characteristics.

Research indicates that incorporating visual feedback during motor skill learning tasks can improve skill acquisition and retention, along with enhancing proprioception^29,30^. Therefore, we hypothesize that the visual feedback component in our structured exercise intervention may significantly impact motor learning and proprioception. Additionally, the inclusion of stretching exercises may provide sustained benefits to participants, contributing to the significant improvement in facial functional measures including resting symmetry, voluntary movement, synkinesis, and HBS grade.

Several underlying mechanisms can be responsible for the significant improvements in multiple outcome measures observed in the experimental group when compared to the conventional group. Firstly, the experimental group intervention aimed to address the imbalance between facial muscles typically observed in BP by targeting both overactive and weakened muscles^31^. Through a combination of unique stretching and specific muscle facilitation techniques, the experimental group achieved a more balanced activation of facial muscles with improved resting asymmetry and voluntary movement (*p* < 0.05 for all measures). Furthermore, the frequency and intensity of the intervention^11,12,32^ may have played a crucial role in promoting faster recovery and greater improvement in facial function. The higher treatment compliance rates observed in the experimental group indicate that participants were more engaged with and adherent to the intervention, potentially leading to more substantial gains in physical function and social interaction, as noted in the FDI scores. Moreover, the use of visual feedback, particularly in front of a mirror during facial exercises, may have enhanced the effectiveness of the facial stretching and structured exercise intervention.

This study has several limitations that warrant consideration when interpreting the results and implications for future research. Firstly, the absence of electromyography (EMG) limits our ability to assess the neuromuscular activity and coordination of facial muscles during interventions. EMG could provide valuable insights into the underlying mechanisms of the observed improvements in facial symmetry and function. Understanding the muscle activation patterns and neuromuscular coordination could offer a more comprehensive understanding of the efficacy of the novel stretching and exercise program compared to conventional approaches. Secondly, the sample size of the current study is relatively small and may affect the generalizability of the findings. While the study adhered to rigorous inclusion and exclusion criteria, larger randomized controlled trials are needed to validate the effectiveness of the proposed intervention across diverse populations with BP. A larger sample size would also allow for subgroup analyses to identify potential predictors of treatment response and optimize individualized treatment protocols. Thirdly, the lack of long-term follow-up data is another limitation of this study. Long term follow-up of observed improvements in facial symmetry and function would provide valuable insights into the durability of the intervention effects. Therefore, future studies should assess the cost-effectiveness and efficacy of combining facial stretching with structured exercises, compared to other therapies, with a larger sample size, EMG measures and long-term follow-up of outcome measures.

### Supplementary Information

Supplementary Information.

## Data Availability

Data sheet and appendix are available from 10.17605/OSF.IO/UVW89.
