# Supplementary material for: Effectiveness of novel facial stretching with structured exercise versus conventional exercise for Bell’s palsy: a single-blinded randomized clinical trial
Source: Sci Rep. 2024 Jun 10;14:13266. doi: 10.1038/s41598-024-64046-z (PMC11164989; doi:10.1038/s41598-024-64046-z)
Supplement: Supplementary file 1 — Supplementary Information. [file 41598_2024_64046_MOESM1_ESM.docx]

**APPENDIX**

**TREATMENT PROTOCOL FOR THE EXPERIMENTAL GROUP- FACIAL STRETCHING AND STRUCTURED EXERCISE PROGRAM**

The experimental group received the below mentioned exercise regimen that consisted of facial stretching exercises followed by the structured facial exercise program. The details regarding the stretching exercises and the structured facial exercises are as follows.

**Stretching Exercises:** There are four stretching exercise components. Among them, three are performed on the unaffected side by the therapist or the bystander. The therapist will demonstrate the stretching techniques during the first few sessions with adequate instructions. Following which the bystander will be advised to demonstrate the techniques on the patient and once satisfactory the bystander will be asked to continue the exercises as instructed. The last component will be performed by the patient himself on the affected side.

Each component should be repeated 10 times with a stretch hold of a minimum 10 to a maximum of 15 seconds. The stretching components should be repeated 4-5 times a day. The starting point of the three components performed by the therapist or bystander begins near the tragus of the ear (near external auditory meatus) and should be directed horizontally or obliquely across the face (as depicted in the pictures below). The pad of the thumb should be used for the stretching. While stretching, an adequate amount of pressure using the pulp of both thumbs has to be applied over the soft tissue structures of the face and directed towards the midline.

The components are as follows:

1. Starting from the tragus of the ear stretch horizontally across the nasolabial fold and the upper lip (Figure 1A).
2. Starting from the tragus of the ear, stretch obliquely across the upper and lower lips

(Figure 1B).

3. Starting from the tragus of the ear, stretch towards the chin (Figure 1C).

4. Self-stretching on the affected side of the face obliquely from the inferomedial aspect towards the inferolateral aspect of the eyebrow (Figure 1D).

**Structured Facial Exercises:**

Prior to the strengthening exercises, a traditional Strength-Duration (SD) curve test or a Faradic-Galvanic (FG) test to be performed to note the electrical reaction response of facial muscles. If the facial muscles are responding to faradic current (even bare minimal contraction to the best tolerable intensity), the treatment of choice should be faradic current itself. Faradic current has to be given for the facial nerve trunk and its motor nerve branches (two sets of 30 contractions per daily session). Figure 2 depicts the facial motor nerve main trunk and branches for Faradic stimulation. For those subjects who do not respond to faradic current, galvanic stimulation (interrupted direct current of 300ms pulse duration) for the facial muscles (two sets consisting of 30 muscle contractions per set) should be the treatment of choice. Unlike the stretching and facial exercises, the electrical stimulation is performed only once a day.

Post facial stretching exercises and electrical stimulation, facial exercises should be given for the weaker facial muscles of the affected side. All the facial exercises must be done using a visual feedback (preferably, in front of a mirror). The facial exercises taught to the patient should be repeated a minimum of four times a day. Each facial exercise component to be repeated a minimum of 5 times to a maximum of 15 times per session.

Facial exercise components:

1. The subject should be made to sit in front of the mirror with the unaffected eye wide open (one or both hands can be used to keep the eye open). While looking towards the mirror, the subject should try his/her level best to close the affected eye, like winking on the affected side (Figure 3A).

2. Eyebrow raise – The therapist must position his/her fingertips slightly above the eyebrows and assist the eyebrows to raise and explain to the patient to hold the position (Figure 3B).

3. The subject is asked to move the nose upwards bilaterally or flare up the nostril unilaterally (Figure 3C).

4. Clench and show the upper teeth- The subject is given instructions to show the upper teeth (Figure 3D).

5. Frowning of the face (Figure 3E). Therapist or bystander may give assistance for the affected side to produce the frowning action.

6. Smiling towards the affected side (Figure 3F).

7. Whistle or Blowing – The subject is asked to perform the action of blowing (Figure 3G).

8. Lip puckering - Ask the subject to pucker the lips.

Throughout the facial strengthening exercises, to avoid overactivity of facial muscles on unaffected side, the patient or therapist should pull the cheeks of the unaffected side forwards and towards the affected side (Figure 3C, 3D, 3F, & 3G). Similarly, for the affected side eyebrow raising, the unaffected side eyebrow could be held downwards to minimize the overactivity of the latter side (Figure 3H). The exercise set should be performed 4 times a day. In case there are no observable minimal facial movements on the affected side during the facial exercises, assistance can be given until observable movements are visible and later should be withdrawn. Once able to voluntarily produce satisfactory-good facial expression movement, manual resistance can be introduced to further improve the strength.

**TREATMENT PROTOCOL FOR CONVENTIONAL GROUP**

The traditional exercise is given after the electrical stimulation (same regimen as mentioned in the treatment protocol for the experimental group). The traditional exercise regimen is in line with the North East London Foundation Trust (NELFT) - National Health Service Trust (NHS) information leaflet. The exercise set should be performed 4 times a day. The exercises need to be performed in front of the mirror and the components are as follows:

1. Eyebrows raise - Position fingertips slightly above the eyebrows and assist the eyebrows to rise 3 times. On the third attempt hold the position
2. Frown - With fingers positioned as above, gently assist movement as you attempt to frown.
3. Eye closure - Place fingers above and below the eye socket – stretch to a fully open position and then pull the fingers together to close the eye.
4. Flexible cheeks - Place 2 fingers on the front teeth, run them along to the back of the mouth, pull and stretch the cheeks and run fingers to the bottom of the gums.
5. Cheeks blowing - Seal lips, using fingers to assist if required, and blow the cheeks out as symmetrically as possible.
6. Straight smile - With the assistance of your fingers on each side of the mouth, move the corners of your mouth outwards as if saying 'ee'.
7. Full smile - With your fingers on your cheeks, curl the corners of your mouth upwards. Complete each exercise 5 times, four to five times a day.


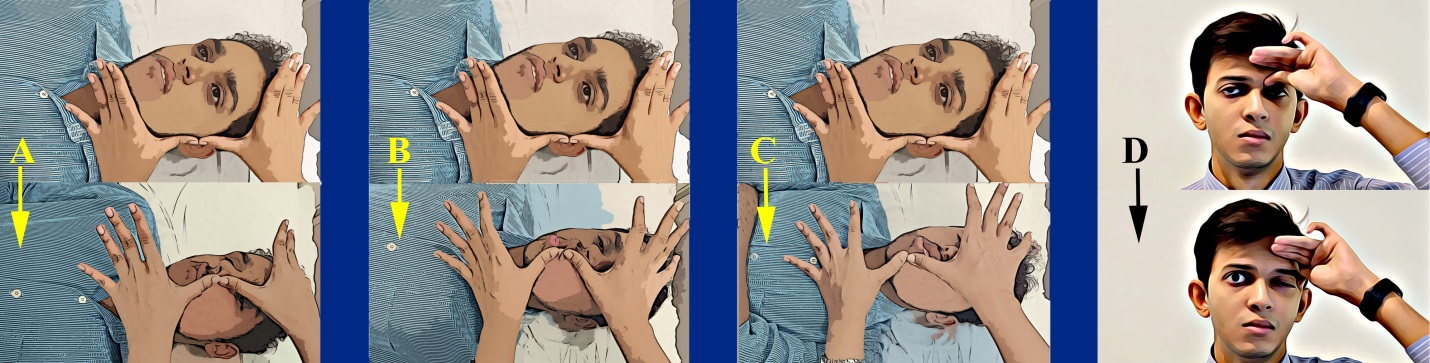


Animated photograph of models with permission

**Figure 1. Illustrations the facial stretching exercises**


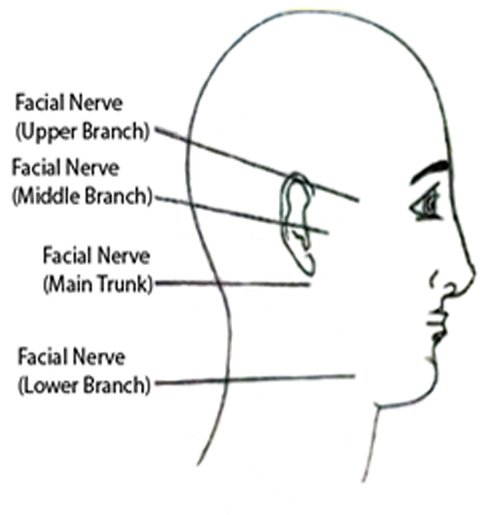


**Figure 2. Facial motor nerve main trunk and branches- for faradic stimulation**


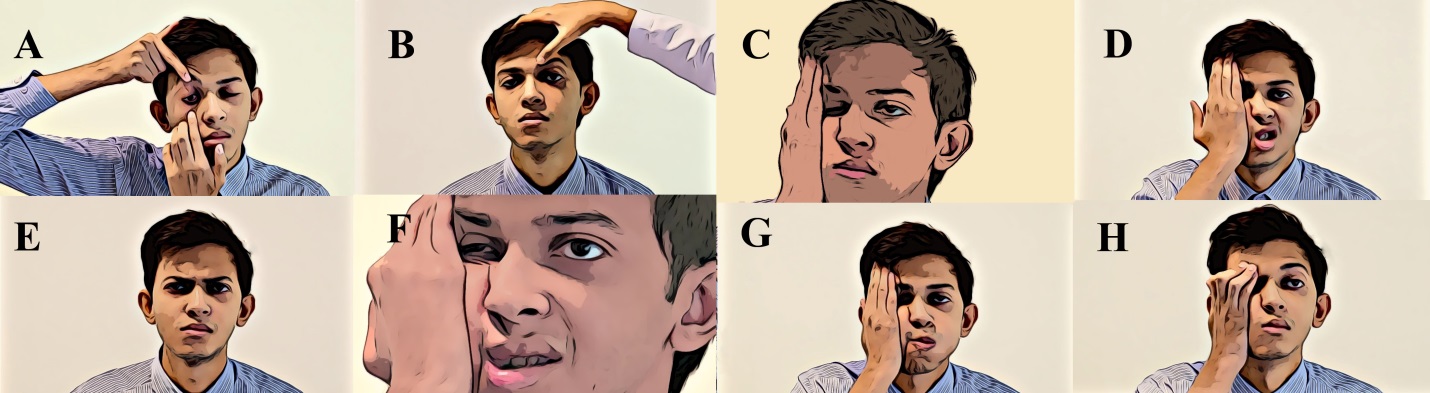


Animated photograph of model with permission

**Figure 3. Depictions of facial exercises**
